# Functional Polymers and Polymeric Materials From Renewable Alpha-Unsaturated Gamma-Butyrolactones

**DOI:** 10.3389/fchem.2019.00845

**Published:** 2019-12-13

**Authors:** Jozef Kollár, Martin Danko, Falko Pippig, Jaroslav Mosnáček

**Affiliations:** ^1^Department of Synthesis and Characterization of Polymers, Polymer Institute, Slovak Academy of Sciences, Bratislava, Slovakia; ^2^Centre for Advanced Materials Application, Slovak Academy of Sciences, Bratislava, Slovakia

**Keywords:** Tulipalin A, sustainability, polyesters, ATRP, polyamidoamines, methacrylates

## Abstract

Sustainable chemistry requires application of green processes and often starting materials originate from renewable resources. Biomass-derived monomers based on five-membered γ-butyrolactone ring represent suitable candidates to replace sources of fossil origin. α-Methylene-γ-butyrolactone, β-hydroxy-α-methylene-γ-butyrolactone, and β- and γ-methyl-α-methylene-γ-butyrolactones bearing exocyclic double bond are available directly by isolation from plants or derived from itaconic or levulinic acids available from biomass feedstock. Exocyclic double bond with structural similarity with methacrylates is highly reactive in chain-growth polymerization. Reaction involves the linking of monomer molecules through vinyl double bonds in the presence of initiators typical for radical, anionic, zwitterionic, group-transfer, organocatalytic, and coordination polymerizations. The formed polymers containing pendant ring are characterized by high glass transition temperature (*T*_g_ > 195°C) and render decent heat, weathering, scratch, and solvent resistance. The monomers can also be hydrolyzed to open the lactone ring and form water-soluble monomers. Subsequent radical copolymerization in the presence of cross-linker can yield to hydrogels with superior degree of swelling and highly tunable characteristics, depending on the external stimuli. The five-membered lactone ring allows copolymerization of these compounds by ring opening polymerization to provide polyesters with preserved methylene functionality. In addition, both the lactone ring and the methylene double bond can be attacked by amines. Polyaddition with di- or multi-amines leads to functional poly(amidoamines) with properties tunable by structure of the amines. In this mini-review, we summarize the synthetic procedures for preparation of polymeric materials with interesting properties, including thermoplastic elastomers, acrylic latexes, stimuli-sensitive superabsorbent hydrogels, functional biocompatible polyesters, and poly(amidoamines).

## Introduction

**Figure 1:**
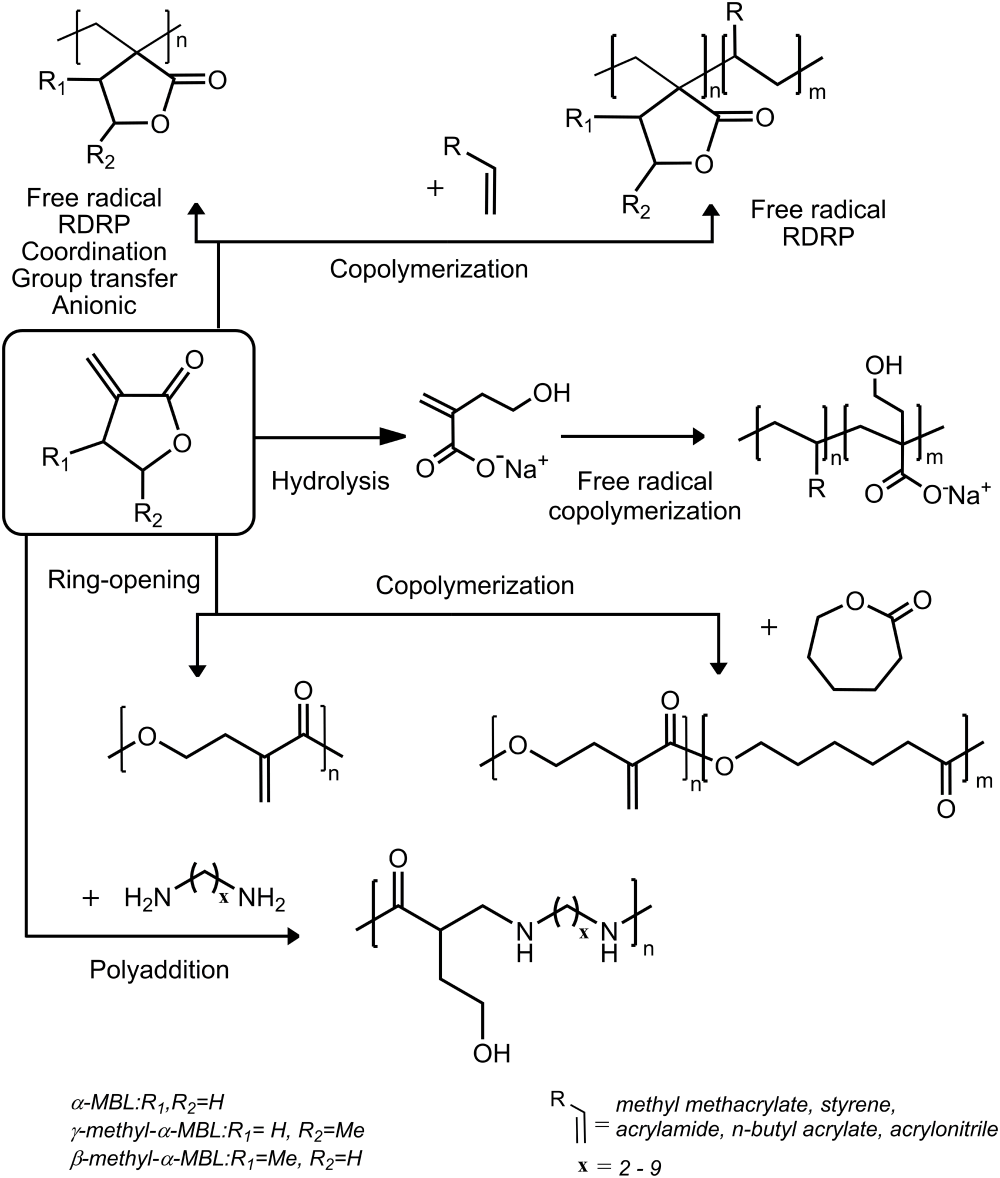
A scheme of polymerization routes for preparation of (co)polymers from MBL derivatives.

The research in the field of renewable or sustainable materials gained increased attention mainly due to the examining the possibility to replace petroleum-based raw materials as polymer materials for large commodity and specialty chemical markets (Vikas, 2012). From this point of view, polylactides and poly(hydroxy-butyrate)s available from biomass-based lactic acid (lactide) or from bacterial biosynthesis as degradable polyesters have been studied for a longer time. On the contrary, the group of unsaturated lactones were significantly less extensively studied so far. Their advantage, however, is that they carry two different functional moieties, namely, vinyl and lactone, in one molecule, while both of them are polymerizable. Thus, these monomers can be taken as replacement of (meth)acrylates in vinyl-addition polymerization or as (co)monomers for preparation of degradable polyesters employing lactone ring-opening polymerization (ROP) (Figure 1). Probably the highest potential can be seen for family of unsaturated γ-butyrolactone-based monomers such as: α-methylene-γ-butyrolactone (MBL), β-hydroxy-α-methylene-γ-butyrolactone (H-MBL), β- and γ-methyl-α-methylene-γ-butyrolactone (β-MMBL, γ-MMBL), and angelica lactones (α- and β-AL). While MBL and H-MBL can be isolated from the tulips (Hoffmann and Rabe, 1985; Kitson et al., 2009), MMBLs and angelica lactones can be derived from itaconic or levulinic acids available from biomass feed stock (Leonard, 1957; Chen et al., 2009; Potvin et al., 2011; Gowda and Chen, 2014). The main advantage of these monomers is in enhanced functionality of the final polymers bearing either pendant double bond, pendant lactone ring, or other pendant substituents allowing for various post-functionalizations or employing combination with other polymerization technique for production of desired materials. The applicability of these monomers is enhanced by the fact that MBL structural unit as a part of various sesquiterpenoids exhibits multiple biological properties, including antibacterial, cytotoxic, anti-inflammatory, antioxidant, allergenic, and antimicrobial activity (Wu et al., 2016). Research in the field of versatile synthetic routes for their synthesis (Brenna et al., 2017) together with relatively easy chemical recyclability of the polyesters, prepared by ROP, toward its monomers (Hong and Chen, 2016b) expands the applicability of this type of materials.

## Vinyl Addition Polymerizations of MBL Derivatives

The most studies of MBL derivatives describe their polymerization exclusively via vinyl addition of exo-methylene double bond without ring opening of lactone ring using various polymerization techniques, including free radical (Akkapeddi, 1979a), reversible deactivation (Mosnácek and Matyjaszewski, 2008), coordination (Miyake et al., 2010a), group transfer (Miyake et al., 2010b), or anionic polymerization (Hu et al., 2011; Figure 1).

### Free Radical Polymerization of MBL Derivatives

The first brief description of free radical polymerization of the unsubstituted MBL, known also as a Tulipalin A, was reported by McGraw in a patent in 1953 (McGraw, 1953) and subsequently more detailed by Akkapeddi (Akkapeddi, 1979a). The poly(α-methylene-γ-butyrolactone) (PMBL) prepared by radical polymerization is atactic with slight preponderance of syndiotactic placements. This amorphous polymer has interestingly high *T*_g_ of 195°C and is thermally stable till ~320°C. The polymer shows poor solubility in common organic solvents; the polymers are soluble in DMF and DMSO. Structurally, MBL represents the cyclic analog of methyl methacrylate, but exhibits higher reactivity in free radical polymerizations due to the nearly planar lactone ring and consequently better resonance stabilization of radical species (Ueda et al., 1982).

Early study of homopolymerization of MBL derivative, such as γ-MMBL was published by Suenaga (Suenaga et al., 1984). Chirality of the derivative influences final polymer properties. Unlinke PMBL, the racemic poly(γ-MMBL) renders good solubility in common organic solvents, such as acetone, acetonitrile, chloroform, DMSO, and DMF. On the contrary, the polymers obtained from chiral lactone showed poor solubility in common organic solvents. The reason is formation of highly isotactic polymer. This indicates that monomer placement is dictated by the chirality of the monomer due to less steric approach. *T*_g_ of poly(γ-MMBL) varied from 210 to 220°C regardless of the chirality. γ-MMBL was polymerized also by emulsifier-free miniemulsion polymerization carried out via homogeneous nucleation mechanism, while stable nanoparticles with the size from 60 to 200 nm were obtained (Qi et al., 2008). In 2003, Pittmann (Pittman and Lee, 2003) reported homopolymerization of another MBL derivative, β-MMBL. The resulting poly(β-MMBL) was readily soluble in DMSO and in acetonitrile; however, no *T*_g_ was reported.

Excellent transparency, heat, solvent and scratch resistance of PMBL homopolymers offer efficient platform for the development of more complex MBL-containing polymeric materials. Therefore, the free-radical copolymerizations of MBL monomer with MMA, styrene, acrylamide, acrylonitrile, and vinylene carbonate (Akkapeddi, 1979b; Ueda et al., 1982) and halogenated styrenes (Trumbo, 1995) were studied as well. The copolymerization reactivity ratios were higher for MBL compared to the comonomers. Similarly, higher copolymerization reactivity ratios were found also for MBL derivatives, γ-MMBL and β-MMBL, during their copolymerization with styrene and MMA (Pittman and Lee, 2003; Cockburn et al., 2010) and *n*-butyl acrylate (BA) (Cockburn et al., 2011). In addition to batch copolymerizations, a γ-MMBL was successfully used also in copolymerization with styrene in miniemulsion (Qi et al., 2008) and using stabilizer-free precipitation either in water (Qi et al., 2008) or in isoamyl acetate (Ramram et al., 2016) to form polymeric particles of size in the range of 50–200 or 600–2600 nm, respectively. In the precipitation polymerization performed in isoamyl acetate, the study of the mechanism of the poly(MBL-co-St) particles formation was found to be similar to that of conventional precipitation polymerization in water; e.g., after a short nucleation stage, the amount of polymer particles remained constant and the newly formed polymer chains are captured by particles progressively growing with polymerization time. The difference was that the particles started to precipitate at size as big as ~600 nm. Thus, the size of the nanoparticles can be modulated based on selection of media used for precipitation polymerization.

Properties of PMBL were utilized in dental resin formulations, where MBL was copolymerized with 2,2-bis-[4-(2-hydroxy-3-methacryloxypropoxy)phenylene]propane (Stansbury and Antonucci, 1992). It was found that MBL has a beneficial effect on diametral tensile strength and degree of cure of resin formulations.

### Reversible Deactivation Radical Polymerizations of MBL Derivatives

Development of reversible-deactivation radical polymerizations (RDRP) together with increasing demand on renewable materials opened a new era for synthesis of well-defined functional materials from renewable monomers such as MBL derivatives. First precise polymerization of MBL with control over molecular characteristics was performed by Mosnácek and Matyjaszewski (2008). The MBL was polymerized via atom transfer radical polymerization (ATRP) technique providing polymer with predeterminable ${\bar{M}}_{n}$ and narrow dispersity (Ð = 1.09). Recently, the polymerization was optimized using a more environmentally friendly ATRP technique, photochemically induced ATRP (photoATRP), while only ppm amounts of the catalyst were used and the polymerization was performed at room temperature without necessity to remove oxygen from the polymerization mixture (Zain et al., 2019). High-density semirigid PMBL brushes were also produced by surface-initiated atom transfer radical polymerization of MBL (Higaki et al., 2012). The PMBL brushes exhibited stable friction coefficient with great potential for practical scratch resistant applications. RDRP techniques, namely, miniemulsion reversible addition-fragmentation chain transfer (RAFT) (Qi et al., 2008) and RAFT ab initio emulsion (Xu et al., 2013) polymerizations were used also for preparation of poly(γ-MMBL) (nano)particles.

Livingness of the ATRP technique was used for preparation of well-defined triblock copolymers containing middle soft poly(*n*-butyl acrylate) (PBA) and outer hard PMBL (Mosnáček et al., 2009). The resulting thermoplastic material could be suitable for high-temperature applications. The low solubility a quite low elongation at break and tensile strength of the triblock copolymers were significantly improved by preparation of star-like polymers with PBA-*b*-PMBL arms, while high thermal stability was retained (Juhari et al., 2010).

### Anionic and Coordination Polymerization of MBL Derivatives

Living anionic polymerization of MBL was investigated by Akkapeddi (1979a) at either 0 or −78°C. He found an increase in isotacticity of the PMBL up to 75% with a decrease of the polymerization temperature. However, the degree of isotacticity was still not high enough to provide crystallization.

Recently, living polymerization of MBL and γ-MMBL was investigated using ambiphilic silicon propagating species (Scheme S1) (Miyake et al., 2010b). Polymerizations followed the zero-order kinetics, while for γ-MMBL, quantitative conversions were obtained in 10 min and narrow dispersity polymer was formed. The authors however did not discuss the significantly higher molar masses of the polymer compared to theoretical ones. For polymerization of MBL, low conversions and bimodal distributions of molar masses were obtained due to insolubility of the formed polymer in dichlormethane used as a solvent. Livingness of the system was confirmed by preparation of block copolymers.

Coordination polymerizations of MBL, γ-MMBL, and β-MMBL were investigated by discrete half-sandwich rare-earth metal dialkyl catalysts (Hu et al., 2012). The first-order kinetics for this type of catalysts were found, while almost quantitative conversions were obtained in very short time—in some experiments even below 1 min. However, the control over the molecular characteristics was poor, leading to higher molar masses (low initiatior efficiency) and broad dispersity, probably due to the high activity of the catalyst. Interestingly, unlike for MBL and γ-MMBL, in the case of β-MMBL, the polymerization provided Highly isotactic polymer, insoluble in common organic solvents, with extremely high *T*_g_ of 290°C. Some other coordination polymerizations of MBL derivatives with non-metallocene benzyl complexes (Gowda and Chen, 2013) or lanthanide and early metal catalysts (Miyake et al., 2010a) were described but with no improvement in control over the molecular characteristics. Improvement was found using C2-ligated zirconocenenium catalysts (Scheme S2) (Chen et al., 2012) providing poly(γ-MMBL) with narrow dispersity and *M*_n_ close to theoretical ones. In none of these works, however, was livingness of the coordination polymerization investigated.

### Copolymerization of Hydrolyzed MBL Derivatives

Recently, it was demonstrated that hydrolysis of MBL (Kollár et al., 2016) and γ-MMBL (Luk et al., 2017) monomers with sodium hydroxide proceeds rapidly to form water-soluble sodium 4-hydroxy-2-methylene butanoate (SHMB) and sodium 4-hydroxy-4-methyl-2-methylene butanoate (SHMeMB), respectively. Radical homopolymerization was very slow; however, they copolymerized readily with acrylamide (AM) (Figure 1). When the copolymerization was performed in the presence of cross-linker, superabsorbent hydrogels were obtained. The hydrogels with higher SHMB or SHMeMB content possessed significantly higher water absorption capacity than common commercially available superabsorbent hydrogels based on copolymers of acrylic acid with AM. The mechanical characteristics of hydrogels were shown to be also highly tunable. Hydrogels with higher content of AM rendered better mechanical properties and could be suitable for cell culturing or tissue engineering, since they were found to be non-cytotoxic materials. In addition, effect of various stimuli, such as pH, ionic strength, and temperature were investigated to prove stimuli-responsive behavior of the hydrogels (Kollár et al., 2019). For agricultural purposes, the phytotoxic properties of SHMB/AM hydrogels were tested and compared with conventional and commonly used hydrogels (Rychter et al., 2019). Among tested materials, SHMB-based hydrogels were found to be non-toxic with even slightly positive effect on growth of green parts of plants.

## Ring opening (co)polymerization of MBL

Biocompatibility and biodegradability are the properties that make the aliphatic polyesters interesting for various applications (Nair and Laurencin, 2007). Enhanced functionality enables these polymers to be finely tuned toward the specific applications. The renaissance for ROP of γ-butyrolactone (BL)-based monomers as “non-polymerizable” (correctly said hardly polymerizable) lactone family brought recently highly active lanthanide complexes (Hong and Chen, 2016a), organo-catalysts as super-base ^t^Bu_4_-P (Hong and Chen, 2016b), and urea catalysts (Lin et al., 2018). These systems allowed initiation of BL polymerization at lower temperatures, i.e., in the range from RT to −40°C, thus decreasing probability of side reactions such as transesterification or depolymerization. This also allowed synthesis of high molar mass poly(γ-butyrolactone) (PBL) homopolymer, as a model for functional BL derivatives, at ambient pressure conditions. Approaches utilizing microbial synthesis reported isolation of high molar mass PBL with ${\bar{M}}_{n}$ ~ 10^6^ g mol^−1^ (Moore et al., 2005).

In the case of MBL, the number of examples of its polymerization using ROP is very limited. Up to now, only four works deal with homopolymerization or copolymerization of MBL with other lactones. The first work appeared in 2010 by Zhou et al. (2010), describing copolymerization of MBL with ε-caprolactone (CL) (Figure 1). Common catalysts for ROP under the proposed coordination–insertion mechanism such as tin 2-ethyl hexanoate [Sn(Oct)_2_], titanium(IV) n-butoxide, or Novozym 425 were described to be not active enough to copolymerize MBL. The polymerization was however successful, employing bismuth(III) trifluoromethanesulfonate at 130°C. Copolymers with higher MBL/CL ratio were obtained in moderate yield, their ${\bar{M}}_{n}$ values did not exceed 17 kg mol^−1^, and the dispersity values did not drop below 1.5, indicating low control over the molecular characteristics. The formed polyester with incorporated *exo*-vinylidene group of MBL was subsequently used for cross-linking by free radical copolymerization with methacrylates. These bicomponent cross-linked networks could be applied as material with shape-memory effect. Presence of any PMBL side product, which could be formed by spontaneous thermally initiated radical polymerization via vinyl bond, was not considered by these authors. On the other hand, Hong repeated ROP of MBL under the same conditions and observed formation of the PMBL side product due to high polymerization temperature (Hong and Chen, 2014). The same authors have shown that more active lanthanum-based coordination catalysts are more suitable for preparation of copolyesters of MBL with CL. Due to the combination of the high Lewis acidity and coordination number of the Ln center (desirable for monomer coordination and activation) and the high nucleophilicity of the ligand (desirable for chain initiation), the catalysts such as La[N(SiMe_3_)_2_]_3_, Sm[N(SiMe_3_)_2_]_3_, Nd[N(SiMe_3_)_2_]_3_, and Y(CH_2_SiMe_3_)_3_(THF)_2_ were suitable for ROP copolymerization under the coordination–insertion mechanism. Obtained polyesters had ${\bar{M}}_{n}$ in the range of 20–90 kg/mol; however, the dispersity was broader than 1.5, showing that the process is less selective and thus less controlled from the point of view of the molecular characteristics. The formation of side product, PMBL homopolymer, through polymerization of vinyl bonds has been also observed when the ROP was performed only with MBL without CL at 25°C or for high MBL/CL feed ratios, especially at temperatures over 50°C. However, copolymerization at lower temperatures using the same catalyst and MBL/CL mixture provided only desired copolyester without side product. Depending on the MBL/CL ratio, the content of MBL incorporated into the copolyester chains reached values of 7.7 mol% for 1/1 feed ratio and 20 mol% for 3/1 feed ratio at 25°C. Higher MBL incorporation was achieved at lower temperature (−20°C) while keeping other experimental conditions the same. Application of the strategy for decreasing free Gibb's energy of polymerization by decreasing reaction temperature enabled ring opening of the γ-BL ring in MBL and the authors were able to incorporate up to 40 mol% of MBL into the copolyester.

Further decreasing of the temperature (down to the range of −40 to −60°C) allowed Tang et al. (2016) to prepare, as a first, also homopolyester of MBL fully functionalized with pendant vinyl groups. Thus, polyesters with ${\bar{M}}_{n}$ up to 21 kg mol^−1^ and dispersity over 1.4 were successfully prepared by using yttrium or lanthanum-based catalyst in combination with alcohol. Interestingly, three types of polymeric materials were obtained depending on the La/ROH ratio. For ratio La/ROH −1/3, the polymerization proceeded as ROP under the coordination-insertion mechanism and linear polyester was obtained. For lower content of alcohol (La/ROH −1/2), both ring-opening and vinyl-addition propagations were observed giving cross-linked polymer. Further increase of the La-catalyst content led to PMBL homopolymer formed via addition of vinyl double bonds. The chemo-selectivity of the system controlled by La[N(SiMe_3_)_2_]_3_/ROH ratio, temperature, or concentration may have wide practical applications.

Even though the Ln-based catalyst provides polyesters with high incorporation of functional double bonds along the polymer chain, there were still disadvantages of the catalyst: (a) the polyesters possessed quite broad dispersity due to too high activity of the catalyst; (b) high price; and (c) commercial availability. Very recently, Danko et al. described MBL/CL copolymerization by two mechanisms, namely, coordination–insertion mechanism and monomer-activated mechanism (Danko et al., 2018). In the first case, the linear functional P(MBL-*co*-CL) copolyesters with ${\bar{M}}_{n}$ ~ 15 kg mol^−1^ and 16–25 mol% of MBL content were synthesized by employing tris aluminum isopropoxide [Al(^i^OPr)_3_] as a cheap and commonly available catalyst, while high MBL/CL feed ratio (5–10/1) was used. In addition, high molar mass copolyesters with ${\bar{M}}_{n}$ ~ 25–90 kg mol^−1^ and 3–7 mol% of MBL content were synthesized from 1/1 or lower MBL/CL feed ratio. Dispersity values for all polyesters were kept in the range of 1.2–1.3, confirming good control over the molecular characteristics thanks to the lower activity of the catalyst compared to Ln-based ones. Based on the coordination–insertion mechanism (Scheme S3), the polymers can be initiated only by isopropoxide groups, thus not allowing introduction of functionality at the beginning of the polymer chain or preparation of star polymers. This is however allowed by application of the monomer-activated mechanism (Scheme S4) and was proved by preparation of star-shaped P(MBL-*co*-CL) using metal-free diphenyl phosphine (DPP) catalyst in combination with multi-hydroxyl alcohol as co-catalyst. In order to prove the availability of the pendant double bonds for post-funtionalization and the possibility of the polyester to act as a carrier for biologically active compounds, both thermal and photochemical thiol-ene click reactions of benzothioxanthene fluorophore and *N*-acetyl cysteine, respectively, were successfully reported.

## Polyaddition of MBL with diamines

MBL can undergo two different reactions with amines. It is described that the BL ring can be open by amines under formation of an amides. The aminolysis of the BL ring of γ-valerolactone (γ-VL) toward amides was investigated by Chalid et al. (2012). When the γ-VL was in excess under reaction with diamines, the diamides were formed. Similarly, for a dimer made out of a thiolactone and a butyrolactone ring, functional polymers were formed by polyaddition reactions (Marquardt et al., 2017). It is also well-known that (meth)acrylates can react with amines via Michael addition. Thus, in the case of MBL, both vinyl group and lactone ring can react with amines. Jean et al. published an investigation that deals with the synthesis of branched polyamines from MBL (Jean et al., 2005). It was presented that different *N*-tert-butoxycarbonyl (BOC) protected diamines reacted via Michael addition with the double bond of MBL, while it was possible to add mono-protected as well bi-protected diamines. Our recent results showed that when MBL is reacted with unprotected diamines, the poly(amidoamines) can be prepared (Figure 1). When the polymerization was performed under 100°C, linear polymers were formed. For comparison, so far linear the poly(amidoamine)s were synthesized via reactions of diacrylates with primary (Isca et al., 2016) or secondary (Emilitri et al., 2005) amines or aminoalcohols (Elzes et al., 2016) and others usually possessed (hyper)branched structure (Wang et al., 2005). Therefore, the linear poly(amidoamine)s from MBL can provide a new approach toward synthesis of this class of functional polymers with novel properties worth to be investigated.

## Conclusions

A wide range of functional polymers can be prepared from renewable MBL derivatives thanks to the presence of two different functionalities in their structure. Polymerization through vinyl moieties provides polymers with pendant lactone ring or its hydrolyzed form. Oppositely, when lactone ring is used in ring opening (co)polymerization, hydrolyzable copolyesters with pendant double bonds are prepared. In both cases, the polymers and polymeric materials with novel and interesting properties are formed (Table 1). Postfunctionalization of the MBL-based polymers can provide further variation of the properties. In addition, poly(amidoamine)s, which can be prepared by polyaddition of MBL with diamines, can open space for a novel class of polymers derived from MBL derivatives with wide application potential, similar to diacrylates-based poly(amidoamine)s, which were previously studied for different applications, such as wastewater treatment (Ferruti et al., 2006), fire protection (Manfredi et al., 2018), or paper conservation (Isca et al., 2019). Poly(amidoamine)s showed low cytotoxicity (Li et al., 2016). Therefore, another big class of application of poly(amidoamine)s are in medicine or biotechnology (Hartmann et al., 2006; Magnaghi et al., 2011; Martello et al., 2014; Ndamase et al., 2018). In addition, MBL-based poly(amidoamines) contains additional functional groups, such as secondary amine in the main chain and pendant hydroxyl groups, which can be further used for postfunctionalization and can broaden their application area. Although, nowadays, the availability of MBL monomers is complicated and they are quite expensive; once there are commercial applications of the MBL-based polymers, they can replace various petroleum-based polymers. This can be further supported by the fact that efficient synthesis of γ-MMBL was developed by DuPont, and scale-up to commercial production has already been investigated.

**Table 1:** A summary of properties of polymers from MBL derivatives.

**Polymer**	**Polymerization technique**	**Properties**	**References**
PMBL	Free radical, ATRP, photoATRP, coordination	Amorphous, atactic, *T*_g_ of 195°C, excellent transparency, heat, and solvent resistance (soluble only in DMF and DMSO)	Akkapeddi, 1979a; Mosnácek and Matyjaszewski, 2008; Miyake et al., 2010a; Zain et al., 2019
	Anionic	Isotactic, soluble in DMF, and DMSO	Akkapeddi, 1979a
PMBL brushes	Surface-initiated ATRP	Stable friction coefficient, scratch resistance	Higaki et al., 2012
Poly(γ-MMBL)	Radical, anionic, miniemulsion, group-transfer, coordination	Racemic—good solubility in organic solvents; Chiral—poor solubility in organic solvents; *T*_g_ of 210–220°C	Suenaga et al., 1984; Qi et al., 2008; Hu et al., 2012
Poly(β-MMBL)	Radical	Soluble in DMSO and acetonitrile	Pittman and Lee, 2003
	Coordination	highly isotactic polymer, insoluble in common organic solvents, *T*_g_ of 290°C	Hu et al., 2012
Triblock PMBL-b-PBA-*b*-PMBL or starlike PBA-b-PMBL	ATRP	Properties of thermoplastic elastomers; high temperature stability up to >300°C	Mosnáček et al., 2009; Juhari et al., 2010
AM-co-SHMB and AM-co-SHMeMB networks	Free radical copolymerization of hydrolyzed MBL derivatives	Superabsorbent hydrogels, stimuli sensitive, low cytotoxicity, and low fytotoxicity	Kollár et al., 2016, 2019; Luk et al., 2017; Rychter et al., 2019
Linear or starlike P(MBL-*co*-CL)	Ring opening polymerization	Hydrolyzable, (multi)functional—possible carriers of active compounds through thiol-ene click	Hong and Chen, 2014; Tang et al., 2016; Danko et al., 2018
P(MBL-*co*-CL) gels	Ring opening polymerization with subsequent free radical copolymerization with methacrylates	Hydrolyzable gels, shape-memory	Zhou et al., 2010

## Author Contributions

JK, MD, and FP collected the references and wrote the draft. JM finalized the manuscript.

### Conflict of Interest

The authors declare that the research was conducted in the absence of any commercial or financial relationships that could be construed as a potential conflict of interest.
