# Supplementary figures and images for: Functional Polymers and Polymeric Materials From Renewable Alpha-Unsaturated Gamma-Butyrolactones
Source: Front Chem. 2019 Dec 13;7:845. doi: 10.3389/fchem.2019.00845 (PMC6923188; doi:10.3389/fchem.2019.00845)

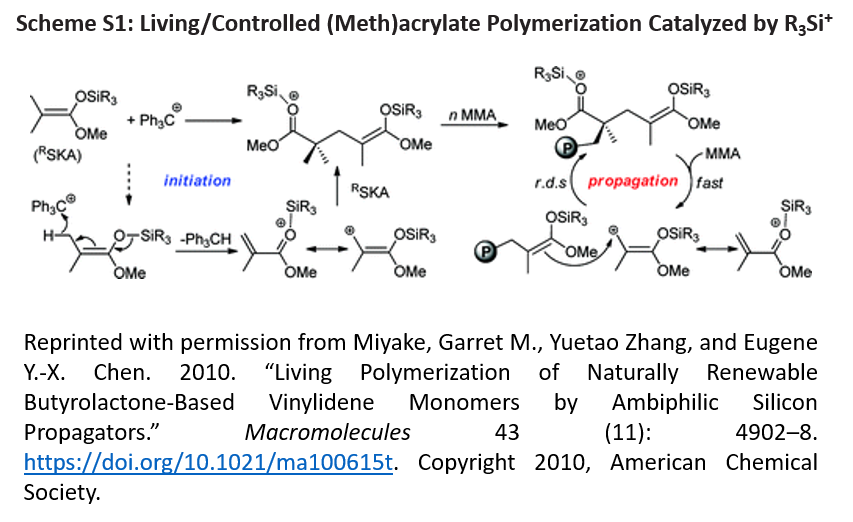

Supplement: Supplementary file 1 [file Image_1.TIF]

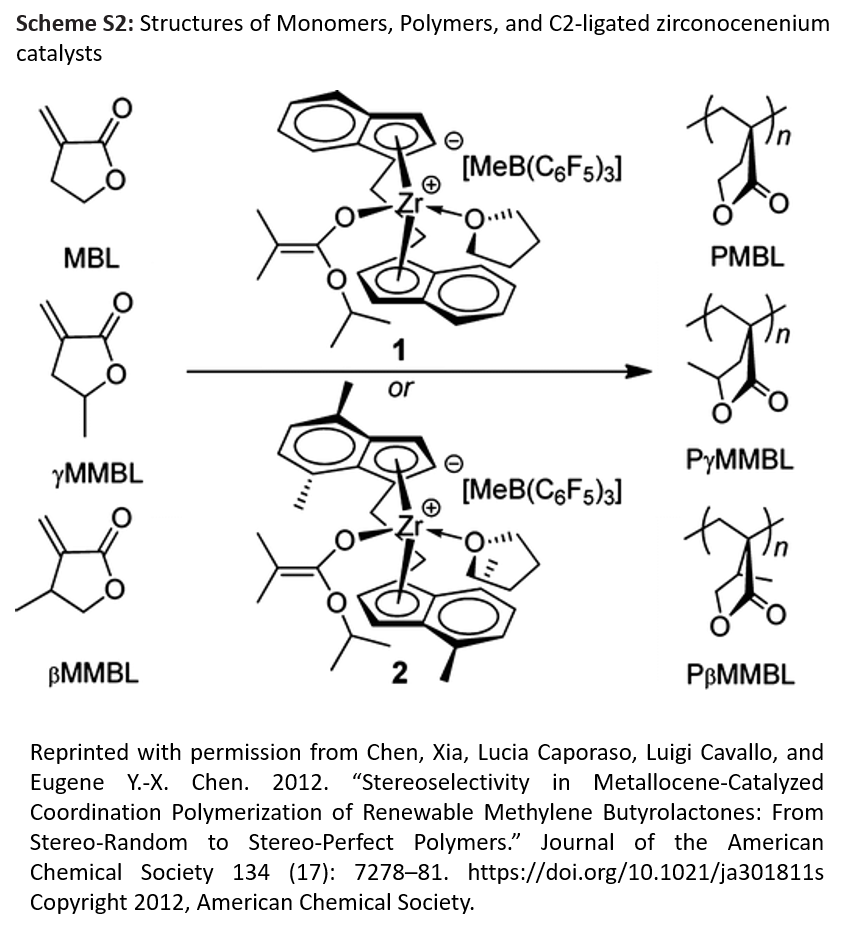

Supplement: Supplementary file 2 [file Image_2.TIF]

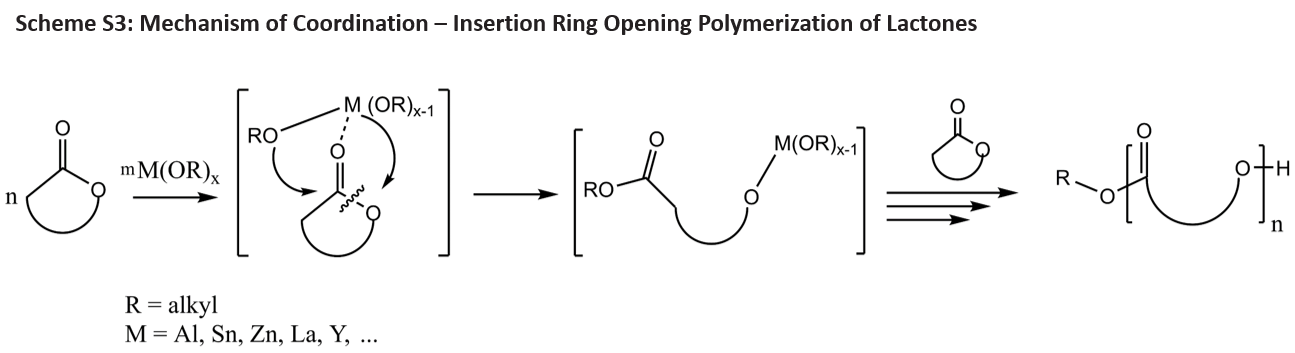

Supplement: Supplementary file 3 [file Image_3.tif]

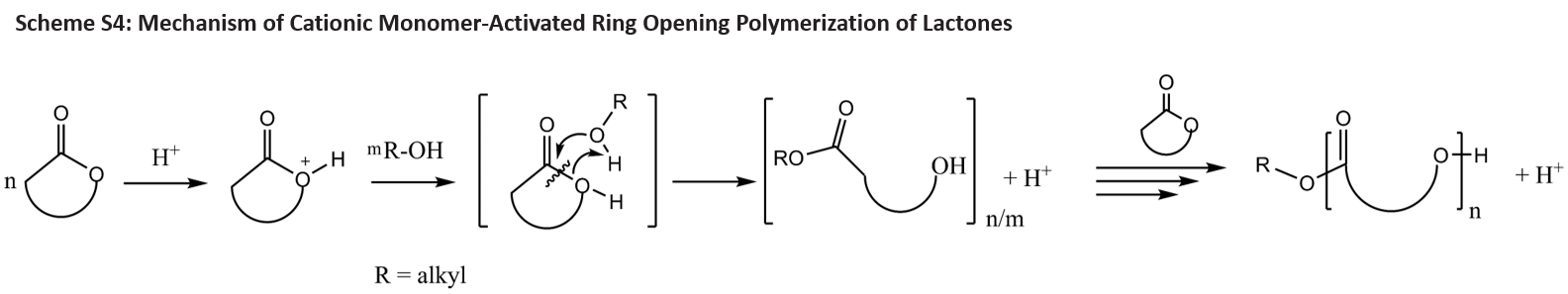

Supplement: Supplementary file 4 [file Image_4.tif]
